# The validity of the subspecies, *Teinopalpus aureus wuyiensis* Lee, from complete mitochondrial genome

**DOI:** 10.1080/23802359.2021.1960214

**Published:** 2021-08-09

**Authors:** Zou Wu, Huang Chao-Bin, Wang Lu, Jiang Meng-na, Zhou Shan-yi, Zhang Jiang-tao, Zeng Ju-ping

**Affiliations:** a Key laboratory of National Forestry and Grass and Administration on Forest Ecosystem Protection and Restoration of Poyang Lake Watershed, College of Forestry, Jiangxi Agricultural University, Nanchang, PR China; b Jiulianshan Forest Ecosystem Observation Station, Longnan, PR China; c Key Laboratory of Ecology of Rare and Endangered Species and Environmental Protection, Ministry of Education, College of Life Science, Guangxi Normal University, Guilin, PR China

**Keywords:** Subspecies validity, molecular evidence, noninvasive sampling, maximum-likelihood phylogenetic tree, intraspecific taxonomy

## Abstract

The complete mitochondrial genome (mtgenome) was determined from the emerged-pupa shell (Noninvasive sampling) of *T. aureus wuyiensis* Lee. It was 15,234 base pairs in length and contained 13 protein-coding genes (PCGs), 2 rRNA genes, 22 tRNA genes, and a control region. By taking *Meandrusa sciron* and *Teinopalpus imperialis* as outgroups, a maximum-likelihood phylogenetic tree was constructed among five geographical populations of *Teinopalpus aureus* based on 13 PCGs and two rRNA genes. Our results showed that the WYS, MHS and PS populations, locating at or closing to Wuyishan Mountain-range, were in one cluster; while the JLS (locating at Nanling Mountain-range) and DYS (locating at Dayaoshan Mountain closing to Nanling Mountain-range) populations belonged to another cluster. It supported well the subspecies of *T. aureus wuyiensis*, and suggested that the genetic relationship between *T. a. guangxiensis* and the nominal subspecies of *T. a. aureus* were closer enough to combine into one subspecies.

The butterfly of golden kaiserihind, *Teinopalpus aureus*, is endemic to tropical and subtropical regions of Asia (Zhou 1994; Igarashi 2001; Wu and Xu 2017), it has been listed as Red Species of IUCN since 1985 (Gimenez Dixon 1996), and also being as one of the First-class of National Key Protected Animals in China since 1989. *Teinopalpus aureus* is one of flagship species to insect-diversity conservation in East Asia (Wang et al. 2020), so it has attracted lots of attention worldwide, from entomologists (e.g., Igarashi 2001; Collard 2007; Zeng et al. 2008) as well as conservation biologists (Wang et al. 2018; Xing et al. 2019), environmental protectors (Li et al. 2013) and (local to national) governments.

*Teinopalpus aureus* was first recorded on a mountain near Lianping (see Figure S1) in northern Guangdong Province, southern China, and it was treated as a subspecies of *T. imperialis*, but soon adjusted as a new species (Mell 1923). Actually, *T. aureus* is a distinct species based on lots of evidences from morphological characteristics (Zhou 1994; Wu and Xu 2017), molecular sequences (Qin et al. 2012; Huang et al. 2015; Huang et al. 2016) and ecological traits (Igarashi 1987; 2001; Zeng et al. 2008, 2012, 2014; Lin et al. 2017) .The second finding of *T. aureus* is occurred in Wuyishan of Fujian (another mountain in southern China, WYS) in 1980s (Zhou 1994), more than 60 years later from the first record. But since then, similar findings are occurred subsequently in Wuzhishan of Hainan island (the third mountain in 1992, WZS), Dayaoshan of Guangxi (the forth mountain in 1994, DYS), Jiulianshan of Jiangxi (a mountain 15 km to Lianping in the north in 1996, JLS, see Figure S1) (Lin et al. 2017), and so on, which thus promote the intraspecific taxonomy of *T. aureus* (Zhou 1994). In view of above findings from different isolated mountains or regions, the subspecies is then identified and named correspondingly, like the nominal subspecies of *T. a. aureus* Mell mainly from Nanling Mountain-range (e.g. JLS population, see Figure S1) (Zhou 1994), *T. a. wuyiensis* Lee mainly from Wuyishan Mountain-range (e.g. WYS population, see Figure S1) (Li and Zhu 1992; Zhou 1994), *T. a. guangxiensis* Chou et Zhou mainly from Guangxi (e.g. DYS population, see Figure S1) (Zhou 1994), and *T. a. hainani* Lee mainly from Wuzhishan Mountain-range (e.g. WZS population), etc. (Li and Zhu 1992; Zhou 1994; Zhong et al. 2000). However, due to (1) the number of specimens used, such as with only female or male specimens, is totally limited in subspecies taxonomy, and then leading to incomplete description in morphology; (2) in particular, there is a lack of other supporting information like genetic, ecological divergence, the intraspecific taxonomy of *T. aureus* is still controversial.

According to the field methods of Zeng (2005), the puparium (emerged-pupa shell) of *T. aureus* were collected from WYS in Huangken of Jianyang city (27.57029N,117.652410E, see Figure S1), Fujian province, China, and then the DNA was extracted by this way of noninvasive sampling (Feinstein 2004; Wang 2001; Storer et al. 2019). The puparium were put into anhydrous ethanol in a tube and preserved at −20 °C in refrigerator. The pupa-shell DNA was extracted by using DNeasy blood and tissue kit (QIAGEN, Valencia, CA), according to the manufacturer's instruction. We used 21 PCR primers to obtain and sequence the mitogenome (GenBank accession number MW900433), the gene annotations were at MITOS (http://mitos2.bioinf.uni-leipzig.de/index.py). The 21 primers were designed according to Qin et al. (2012), Huang et al. (2015) and Huang (2016).

The complete mitochondrial genome of *Teinopalpus aureus wuyiensis* Lee was 15 234 bp (GenBank accession number MW900433) in length, and contained 13 protein-coding genes (PCGs; ATP6, ATP8, COI-III, ND1-6, ND4L, Cytb), two ribosomal RNAs (12S rRNA and 16SrRNA), 22 transfer RNAs, and a putative control region (D-loop). The sequence of the genome and the direction of the protein-coding genes were similar to most reported mitochondrial genes in lepidopteron insects. In the nucleotide composition, it showed high bias toward A and T, with 79.81% in A + T nucleotides. The ATG was the most popular start codon shared with ATP6, CO2, CO3, CYTB, ND4, ND4L, and start codon ATT was shared with ATP8, ND2, ND3, ND5, and ND6. Particularly, the CO1 begins with codon CGA, and the ND1 begins with codon ATG. The conservative stop codon TAA was shared with most of the PCGs except for three genes; ND1, ND3 and ND4L were terminated with stop codon TAG.

To illustrate the phylogenetic relationships of the complete mt genomes from (1) WYS population (MW900433, from the subspecies of *T. a. wuyiensis* Lee) in this study and from other locations (see Figure S1) in GenBank, including (2) JLS population (KP941015, from the subspecies of *T. a. aureus* Mell) (Huang et al. 2015), (3) DYS population (KP941013; from the subspecies of *T. a. guangxiensis* Chou et Zhou) (Huang et al. 2015), (4) PS population (KP941017.1, from the subspecies of *T. a. wuyiensis* Lee) (Huang et al. 2015, Huang 2016), (5) MHS population (KP941016, from the subspecies of *T. a. wuyiensis* Lee) (Huang et al. 2015), the maximum-likelihood (ML) methods with 1000 bootstrap simulations using MEGA software version 7 (Kumar et al. 2016) were performed to construct the phylogenetic tree basing on the tandem nucleotide sequences of 13 PCGs and two ribosomal RNAs (Sun et al. 2020; Wen et al. 2020; Kim et al. 2021), with *Teinopalpus imperialis* Hope (KR018842) as well as *Meandrusa sciron* Leech (LS975123.1) as the outgroups. The ML tree (Figure 1) showed *T. aureus* and the outgroup of *T. imperialis* were independent of each other, and the intraspecific genetic distance (0.0011 ± 0.00067) of *T. aureus* was also significantly shorter (*t* = 54.589, d*f* = 13, *p* < 0.001**)** than that between (0.0212 ± 0.00067) *T. aureus* and *T. imperialis* in *t*-test. In the ML tree, there were two clusters among the above five geographical populations of *T. aureus*, the three WYS, MHS and PS populations were in one cluster, while another two populations, JLS and DYS, were belonged to another cluster (Figure 1). In effect, the ML tree was consistent well with the mountain-range distribution of the five locations geographically, like the WYS, MHS and PS, all locating at or closing to Wuyishan Mountain-range, while the JLS and DYS, both locating at or closing to Nanling Mountain-range (Figure S1). Similarly, the genetic distance within (0.0002 ± 0.00012) the subspecies of *T. a. wuyiensis* (including WYS, MHS, and PS populations) was found significantly shorter (*t* = 6.188, df = 7, *p* < 0.001) than that between (0.0015 ± 0.00033) *T. a. wuyiensis* and *T. a. aureus* (including JLS and DYS populations). Our results supported well the subspecies of *T. aureus wuyiensis*, and suggested that the genetic relationship between *T. a. guangxiensis* and the nominal subspecies of *T. a. aureus* were closer enough to combine into one subspecies (Figure 1). To be noted, continuation of genomic studies is still necessary for clarifying taxonomy of the remaining subspecies.

**Figure 1.**
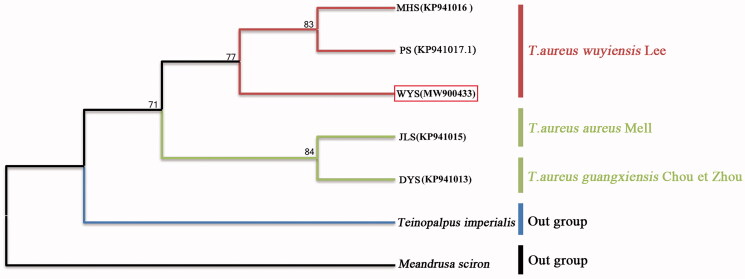
Maximum-likelihood phylogenetic tree for the newly obtained complete mitochondrial genome (WYS – MW900433) of *T. aureus* wuyiensis and the previously determined sequence from GenBank (MHS – KP941016 and PS – KP941017.1 of *T. aureus* wuyiensis; JLS – KP941015 of *T. aureus*; DYS – KP941013 of *T. aureus* guangxiensis). *T. imperialis* (KR018842) and *Meandrusa sciron* (LS975123.1) were used as outgroups. Numbers at nodes were bootstrap supporting values in %.

## Data Availability

The genome sequence data that support the findings of this study are openly available in GenBank of NCBI at (https://www.ncbi.nlm.nih.gov) under the Accession no. MW900433.

## References

[CIT0001] Collard S. 2007. Description d'une nouvelle sous-espece de *Teinopalpus aureus* (Mell 1923) du Laos (Lepidoptera: Papilionidae). Lambillionea. 107:403–406.

[CIT0002] Feinstein J. 2004. DNA sequence from butterfly frass and exuviae. Conserv Genet. 5: 103–104.

[CIT0003] Gimenez Dixon M. 1996. *Teinopalpus aureus*. The IUCN Red List of Threatened Species1996:e.T21557A9301005; [accessed 2021 Apr 5]. 10.2305/IUCN.UK.1996.RLTS.T21557A9301005.en

[CIT0004] Huang CB, Zeng JP, Zhou SY. 2015. Complete mitochondrial genomes of *Teinopalpus imperialis* (Lepidoptera: Papilionidae) and phylogenetic relationships analyses. Mitochondrial DNA. 27(4):1–2. 10.3109/19401736.2015.1060431 26162054

[CIT0005] Huang CB. 2016. Study on phylogenetic of 5 subspecies of Teinopalpus aureus (Lepidoptera: Papilionidae) based on mitochondrial genomes. Guangxi: Guangxi Normal University.

[CIT0006] Igarashi S. 1987. On the life history of *Teinopalpus imperialis* Hope in Northern India and its phylogenetic position in the Papilionidae. Tyȏ to Ga. 38(3):115–151.

[CIT0007] Igarashi S. 2001. Life history of *Teinopalpus aureus* in Vietnam in comparison with that of *T. imperialis*. Butterflies. 30:4–24.

[CIT0008] Kim MJ, Chu M, Park JS, Kim S, Kim I. 2021. Complete mitochondrial genome of the summer heath fritillary butterfly, *Mellicta ambigua* (Lepidoptera: Nymphalidae). Mitochondrial DNA Part B. 6(5):1603–1605. 34027067 10.1080/23802359.2021.1917318 PMC8118395

[CIT0009] Kumar S, Stecher G, Tamura K. 2016. MEGA7: Molecular Evolutionary Genetics Analysis Version 7.0 for bigger datasets. Mol Biol Evol. 33(7):1870–1874. 27004904 10.1093/molbev/msw054 PMC8210823

[CIT0010] Li CL, Zhu BY. 1992. Atlas of Chinese butterflies. Shanghai: Shanghai Far East Publishing Press.

[CIT0011] Li X, Settele J, Schweiger O, Zhang Y, Lu Z, Wang M, Zeng JP. 2013. Evidence-based environmental laws for China. Science. 341(6149):958. 10.1126/science.341.6149.958-a 23990542

[CIT0012] Lin B, Zhu X, Zeng J, Yuan J. 2017. Research on biological characteristics of *Teinopalpus aureus* in Jiulianshan. Forest Res. 30(3):399–408.

[CIT0013] Mell. 1923. Noch unbeschriebene Lepidopteren aus Südchina (2) Dt. Ent. Zs. 1923(2):153–160.

[CIT0014] Qin F, Jiang GF, Zhou SY. 2012. Complete mitochondrial genome of the *Teinopalpus aureus guangxiensis* (Lepidoptera: Papilionidae) and related phylogenetic analyses. Mitochondrial DNA. 23(2):123–125. 22409753 10.3109/19401736.2011.653805

[CIT0015] Storer C, Daniels J, Xiao L, Rossetti K. 2019. Using noninvasive genetic sampling to survey rare butterfly populations. Insects. 10(10):311. 10.3390/insects10100311 PMC6835262 31547512

[CIT0016] Sun Y, Huang H, Zhang X, Xia J, Geng J, Zhang K. 2020. The complete mitochondrial genome of the Papilio paris (Lepidoptera: Papilionidae). Mitochondrial DNA Part B. 5(1):733–735. 33366725 10.1080/23802359.2020.1715281 PMC7748820

[CIT0017] Wang RJ. 2001. Application of faecal DNA analysis in animal ecology. Acta Zool Sin. 6:699–703.

[CIT0018] Wang W, Suman DO, Zhang H, Xu Z, Ma F, Hu S. 2020. Butterfly conservation in China: from science to action. Insects. 11(10):1–29. 10.3390/insects11100661 PMC7600441 32992975

[CIT0019] Wang Z, Huang Y, Luo X, Qin K, Merz R, Zhou S. 2018. Habitat monitoring of an endangered Asian butterfly, *Teinopalpus aureus* (Lepidoptera: Papilionidae) and change in local residents' conservation awareness. J Insect Conserv. 22(5–6):721–729.

[CIT0020] Wen J, Wang J, Wu Y, Cao T, Li Z. 2020. Complete mitochondrial genome of *Hestina assimilis* (Lepidoptera: Nymphalidae). Mitochondrial DNA Part B. 5(2):1269–1271.

[CIT0021] Wu CS, Xu YF. 2017. Butterfly Atlas of China. Fuzhou: Taiwan Strait Publishing and Distribution Group.

[CIT0022] Xing S, Au TF, Dufour PC, Cheng W, Landry Yuan F, Jia F, Vu LV, Wang M, Bonebrake TC. 2019. Conservation of data deficient species under multiple threats: lessons from an iconic tropical butterfly (*Teinopalpus aureus*). Biol Conserv. 234:154–164.

[CIT0023] Zhou Y. 1994. Monographia Rhopalocerorum Sinensium. Henan: Henan Science and Technology Press.

[CIT0024] Zhong XH, Yu DF, Zheng L, He CH, Chen FB, Liu SZ, Wen CJ, Chen GW, Zheng YC, Cai ZX. 2000. Introduction mountain science and researches of Chinese mountains. Sichuan: Sichuan Science and Technology Press.

[CIT0025] Zeng JP. 2005. Studies on biological of *Teinopalpus aureus* Guangxi Chou et zhou. Guangxi: Guangxi Normal University.

[CIT0026] Zeng JP, Lin BZ, Zhu XF, Liu L. 2014. A host plant, *Michelia maudiae*, widespread-distributed in South China for the endangered butterfly of *Teinopalpus aureus*. Acta Agric Univ Jiangxiensis. 36(3):550–555.

[CIT0027] Zeng JP, Zhou SY, Ding J. 2012. Behavior characteristics and habitat adaptabilities of the endangered butterfly *Teinopalpus aureus* in Mount Dayao. Acta Ecol Sin. 32(20):6527–6534.

[CIT0028] Zeng JP, Zhou SY, Luo BT, Qin K, Liang YL. 2008. Morphology and bionomics of the endangered butterfly golden kaiserihind, *Teinopalpus aureus*, in Dayaoshan of Guangxi. Chin Bull Entomol. 45:457–464.

